# Left Atrial Appendage Closure: Between Imaging Precision and Uncertain Clinical Benefit

**DOI:** 10.3390/jcm15187068

**Published:** 2026-09-11

**Authors:** Renè Tezze, Cristina Rizza, Ludovica Rita Vocale, Giorgio Sciaramenti, Alberto Sarti, Giovanni Camaiti, Pierpaolo Cioci, Kristi Hoxha, Isabella Maccaferri, Francesco Paparazzo, Federico Marchini, Gianluca Campo, Rita Pavasini

**Affiliations:** Cardiology Unit, Azienda Ospedaliero-Universitaria di Ferrara, 44124 Ferrara, Italy; rene.tezze@gmail.com (R.T.); cristinarizza.94@gmail.com (C.R.); ludovica.vocale@gmail.com (L.R.V.); giorgio.sciaramenti@gmail.com (G.S.); sarticchi@gmail.com (A.S.); camaitigiovanni@gmail.com (G.C.); pierpaolocioci@gmail.com (P.C.); isabella.maccaferri@edu.unife.it (I.M.); francescopaparazzo1@gmail.com (F.P.); mrcfrc2@unife.it (F.M.); cmpglc@unife.it (G.C.)

**Keywords:** left atrial appendage closure, LAAC, LAA

## Abstract

Left atrial appendage closure has emerged as an established nonpharmacological strategy for stroke prevention in selected patients with atrial fibrillation, particularly those in whom long-term oral anticoagulation is problematic. Its contemporary role, however, is defined by a central tension between procedural precision and clinical uncertainty. Advances in multimodality imaging, including transesophageal echocardiography, cardiac computed tomography, three-dimensional echocardiography, fusion imaging, and emerging computational tools, now allow highly accurate assessment of left atrial appendage anatomy, device sizing, intraprocedural guidance, and postprocedural surveillance. These techniques are essential to minimize peri-device leak, device-related thrombus, and other procedure-related complications in a structure characterized by marked interindividual variability. At the same time, the randomized evidence base remains nuanced. Early warfarin-era trials established the feasibility of LAAC, whereas contemporary comparisons with direct oral anticoagulants and best available medical therapy have yielded more heterogeneous results, reflecting differences in patient selection, comparator regimens, and endpoint design. Taken together, the available data support LAAC as a reasonable option in carefully selected patients at elevated bleeding risk or with limited tolerance for chronic anticoagulation, but not as a universal substitute for oral anticoagulant therapy. Future progress will depend on more refined phenotyping, standardized imaging pathways, and longer-term comparative data to better define which patients derive the greatest net clinical benefit from LAAC.

## 1. Introduction

Atrial fibrillation (AF) represents a major public health burden and is associated with a four- to five-fold increased risk of ischemic stroke. In Europe, 20–30% of ischemic strokes are attributable to AF [1]. Oral anticoagulation (OAC), traditionally with vitamin K antagonists (VKAs) and more recently with direct oral anticoagulants (DOACs), remains the standard of care for stroke prevention; however, its use may be limited by bleeding risk, nonadherence (approximately 30% of patients receiving DOACs are nonadherent at 2 years), cognitive impairment, fall risk, drug interactions, and cost [2]. This treatment gap has fostered the development of nonpharmacological strategies targeting the left atrial appendage (LAA), the source of more than 90% of thrombi in AF [2,3].

Over the last decade, left atrial appendage closure (LAAC) has evolved rapidly, supported by major advances in device technology and multimodality imaging. At the same time, several randomized trials comparing LAAC with medical therapy have yielded partially conflicting results, leaving important questions regarding patient selection and net clinical benefit unanswered [4,5].

The aim of this review is to critically examine the current role of multimodality imaging in LAAC, from patient selection to postprocedural surveillance, and to discuss the evolving clinical evidence supporting this intervention, with particular emphasis on the gap between procedural success and demonstrated clinical benefit.

### 1.1. Literature Search Strategy

A narrative literature search was performed to identify relevant studies addressing multimodality imaging and clinical outcomes in left atrial appendage closure. PubMed/MEDLINE, Embase, and the Cochrane Library were searched using combinations of the terms “left atrial appendage closure”, “left atrial appendage occlusion”, “LAAC”, “LAAO”, “transesophageal echocardiography”, “TEE”, “cardiac computed tomography”, “CCT”, “intracardiac echocardiography”, “ICE”, “multimodality imaging”, “peri-device leak”, “device-related thrombus”, “randomized controlled trial”, and “oral anticoagulation”.

Priority was given to randomized controlled trials, prospective and large observational studies, systematic reviews and meta-analyses, and contemporary international guidelines or expert consensus documents relevant to LAAC. Studies were selected according to their relevance to the main scope of this review, including patient selection, preprocedural planning, intraprocedural guidance, postprocedural imaging surveillance, device-related complications, and comparative clinical outcomes. Additional relevant publications were identified through review of the reference lists of key articles. Only articles published in English were considered. Given the narrative nature of the present review, no formal systematic-review protocol or quantitative meta-analytic approach was applied.

### 1.2. Anatomy and Variability of the LAA

The LAA anatomy is highly variable. It consists of a finger- or stump-like extension of the left atrium (LA) that is unique to each individual, often described as a cardiac “fingerprint”. Its variability (embryology, gross morphology, ostial geometry, number of lobes, and functional properties) carries implications for thromboembolic risk and device-based interventions [6].

The LAA connects to the left atrium through a narrow ostium, which is most often elliptical (68.9%) and is round in only 5.7% of cases [7]. The transition from the trabeculated LAA endocardium to the smooth LA wall is demarcated posteriorly by the ridge adjacent to the left superior pulmonary vein, a key anatomical landmark for device implantation [6,8].

Four principal morphological types of LAA are recognized, classified by the shape and number of lobes [6,9]:

- Chicken wing (~48%): central lobe with a characteristic bend or fold. It is associated with the lowest thromboembolic risk (OR 0.46 for thromboembolism vs. non-chicken wing; 95% CI: 0.36–0.58) [9,10];
- Cactus (~30%): dominant central lobe with secondary lobes extending superiorly and inferiorly (OR 4.08 for stroke vs. chicken wing) [9];
- Windsock (~19%): single dominant elongated lobe (OR 4.8 for stroke vs. chicken wing) [9];
- Cauliflower (~3%): short central lobe with complex internal characteristics and multiple lobes. It is associated with the highest thromboembolic risk (OR 8.02 vs. chicken wing) [9].

Beyond anatomical characterization, LAA emptying velocity is an important predictor of thromboembolism [10,11]. Clinical data suggest that an LAA emptying velocity < 20 cm/s is an independent risk factor for both thrombus formation (RR 2.6) and subsequent embolic events (OR 4.1) [10]. More recent evidence from 649 patients with AF indicates that a threshold of <35 cm/s is independently associated with stroke/TIA (OR 2.18) [11]. In addition, an LAA orifice area > 3.5 cm^2^ confers a six-fold increase in adjusted stroke risk, while an LAA volume > 34 cm^3^ is associated with a seven-fold increase [10].

### 1.3. Implications for Device Selection

The two most widely used FDA-approved devices for LAAC are the Watchman/Watchman FLX (Boston Scientific) and Amulet (Abbott) systems.

LAA anatomical variability directly impacts procedural planning for LAAC. Each device has specific anatomical prerequisites [6,9]:

- A shallow LAA (ostium > 31 mm or length < 17 mm) precludes Watchman implantation, as the nitinol cage requires depth equal to its width;
- Sharp angles (chicken wing morphology) and short neck (<10 mm, cauliflower morphology) can cause difficulty with endocardial device implantation;
- Prominent pectinate muscles, trabeculations, and multilobed anatomy in different planes may complicate device anchoring;
- The Amulet accommodates a slightly larger maximum landing zone (up to 32 mm) than the Watchman.

## 2. Multimodality Imaging for Patient Selection in Left Atrial Appendage Closure: From Traditional Echocardiography to Advanced Imaging

Multimodality imaging is the cornerstone for the planning of percutaneous LAAC, ensuring safe implantation and minimizing complications like device embolization, peri-device leak, tamponade, cardiac perforation and death. To minimize these risks and improve LAA occlusion outcomes, optimal peri-procedural imaging is critical to ensure accurate sizing and safe implantation.

Peri-procedural imaging for patient selection includes different imaging modalities from traditional ones such as transesophageal echocardiography (TEE) and contrast-enhanced cardiac computed tomography (CCT) and emerging ones such as three-dimensional printing (3DP), fusion imaging, AI and deep learning.

They play a crucial role in preprocedural planning excluding the presence of left atrial thrombus, an absolute contraindication to the procedure and a major source of embolic risk and providing a detailed anatomical assessment of the left atrial appendage and of the surrounding structures.

### 2.1. TEE as the Traditional Gold Standard

TEE is the reference imaging modality for patient selection and intraprocedural guidance in LAAC. Its widespread availability, established role in clinical practice, and ability to provide detailed real-time assessment of LAA anatomy have made it the imaging technique of choice in most centers. In addition, unlike CCT, TEE does not require ionizing radiation or iodinated contrast [12]. Preprocedural TEE is primarily performed to exclude LAA thrombus, to characterize LAA morphology and anatomical complexity, and to measure the ostium and landing zone dimensions required for device sizing.

For thrombus exclusion, the LAA should be systematically examined from multiple imaging planes, with particular attention to the distal lobes, where thrombi may be concealed. In addition, dense spontaneous echo contrast (“smoke”) and reduced LAA emptying velocities (<20 cm/s) should be reported, as both are markers of blood stasis and are associated with an increased thromboembolic risk [13,14,15].

### 2.2. Measurement of the Ostium and Landing Zone

Accurate assessment of LAA dimensions, ostium and landing zone is essential for appropriate device selection and procedural success. Pre-procedural TEE should include a comprehensive multiplane evaluation (0°, 45°, 90°, and 135°), preferably at end-systole when appendage dimensions are maximal. Particular attention should be paid to the LAA ostium and landing zone, as inaccurate measurements may results in device undersizing, increasing the risk of peri-device leak and embolization, or excessive oversizing, potentially leading to excessive compression and tissue injury. The LAA ostium is generally defined by a line extending from the circumflex coronary artery region to the ridge between the left superior pulmonary vein and the appendage. Because the ostium is frequently elliptical rather than circular, its dimensions vary across image planes; therefore, the largest measured diameter is generally used for device sizing [12,16].

**Figure 1:**
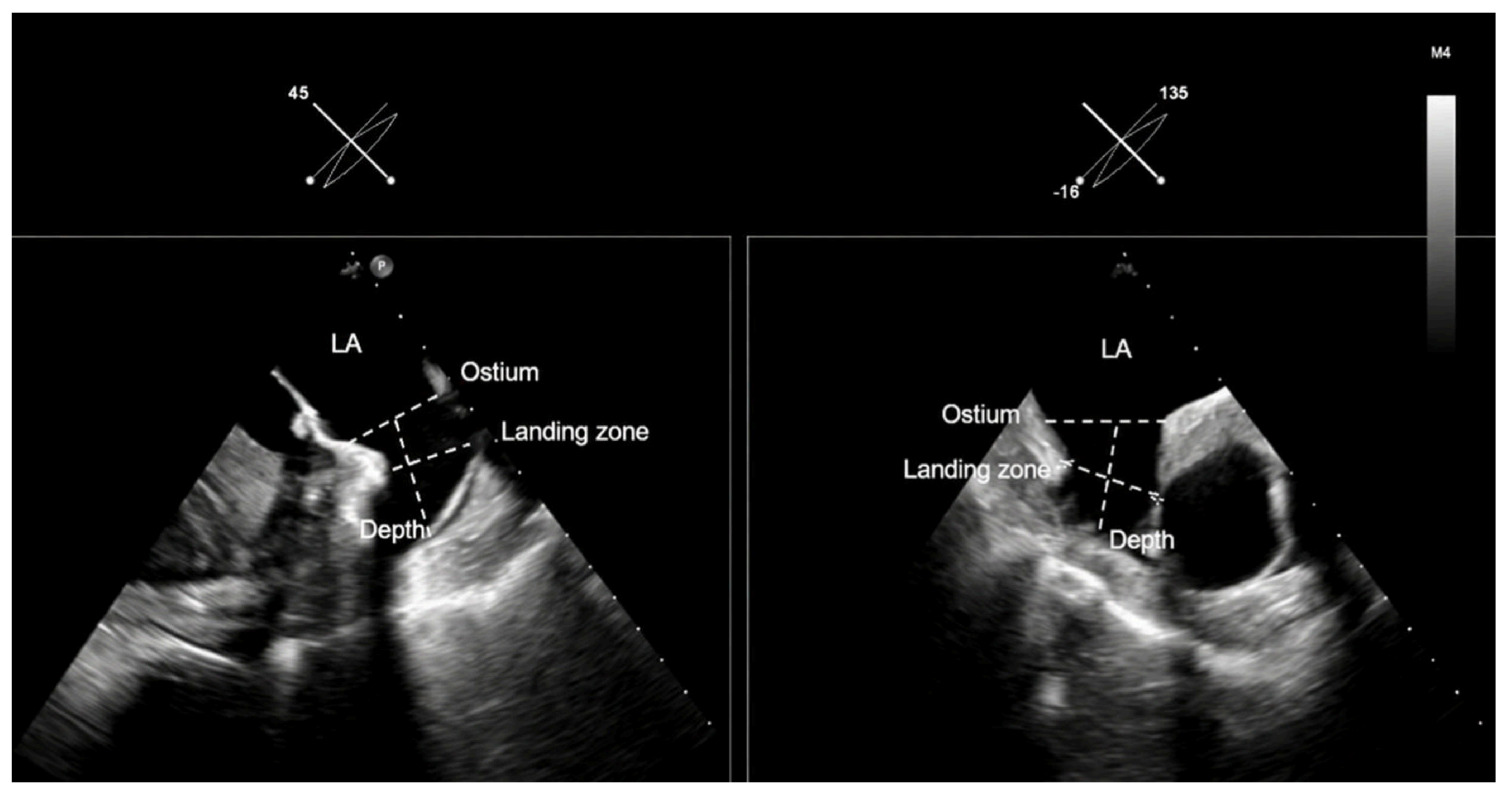
**LAA sizing using 3D TEE**. The LAA orifice diameter and depth are measured at 0°, 45°, 90°, and 135°. The LAA landing zone is measured from the top of the mitral valve annulus or circumflex coronary artery to a point 2 cm below the tip of the left upper pulmonary vein limbus, while depth is measured from the plane of the LAA orifice to the LAA apex. 3D, three-dimensional; LAA, left atrial appendage; TEE, transesophageal echocardiography.

The landing zone corresponds to the segment where the device is anchored and is defined according to the specific device design. For plug-based devices (es. Watchman), measurements are obtained within the proximal appendage at the intended implantation site. For disk/lobe devices (es. Amulet), assessment includes both the anatomic orifice and the more distal anchoring zone [12,16] (Figure 1).

Regardless of the device type, adequate LAA depth must be confirmed to ensure stable deployment and avoid device protrusion into the left atrium. Device-specific criteria should always be considered, as minimum depth requirements differ among available platforms [12,16,17].

### 2.3. Cardiac Computed Tomography: The New Anatomical Gold Standard

CCT has emerged as an alternative imaging modality for preprocedural assessment and planning in patients undergoing LAAC. In addition to providing excellent anatomical characterization of the LAA, CCT demonstrates high diagnostic accuracy for thrombus exclusion, particularly when delayed-phase imaging is employed, with reported sensitivity and specificity approaching 100% and 99%, respectively [18]. Compared with TEE, CCT provides superior spatial resolution and enables comprehensive three-dimensional evaluation of LAA morphology, ostial dimensions, landing zone characteristics, depth, and its relationships with adjacent cardiac structures. This approach may be especially valuable in patients with contraindications to TEE or when esophageal imaging is not feasible, allowing TEE or intracardiac echocardiography (ICE) to be reserved primarily for intraprocedural guidance. Although Yosefy et al. reported comparable measurements of LAA area and volume between CCT and 2D-TEE [19], several subsequent studies have demonstrated that CCT-derived measurements are generally larger, likely reflecting a more accurate characterization of the frequently elliptical anatomy of the LAA ostium and landing zone. Consequently, CCT-based sizing has been associated with improved device selection and lower rates of device-related complications, including peri-device leak (PDL) and device embolization [20,21].

**Figure 2:**
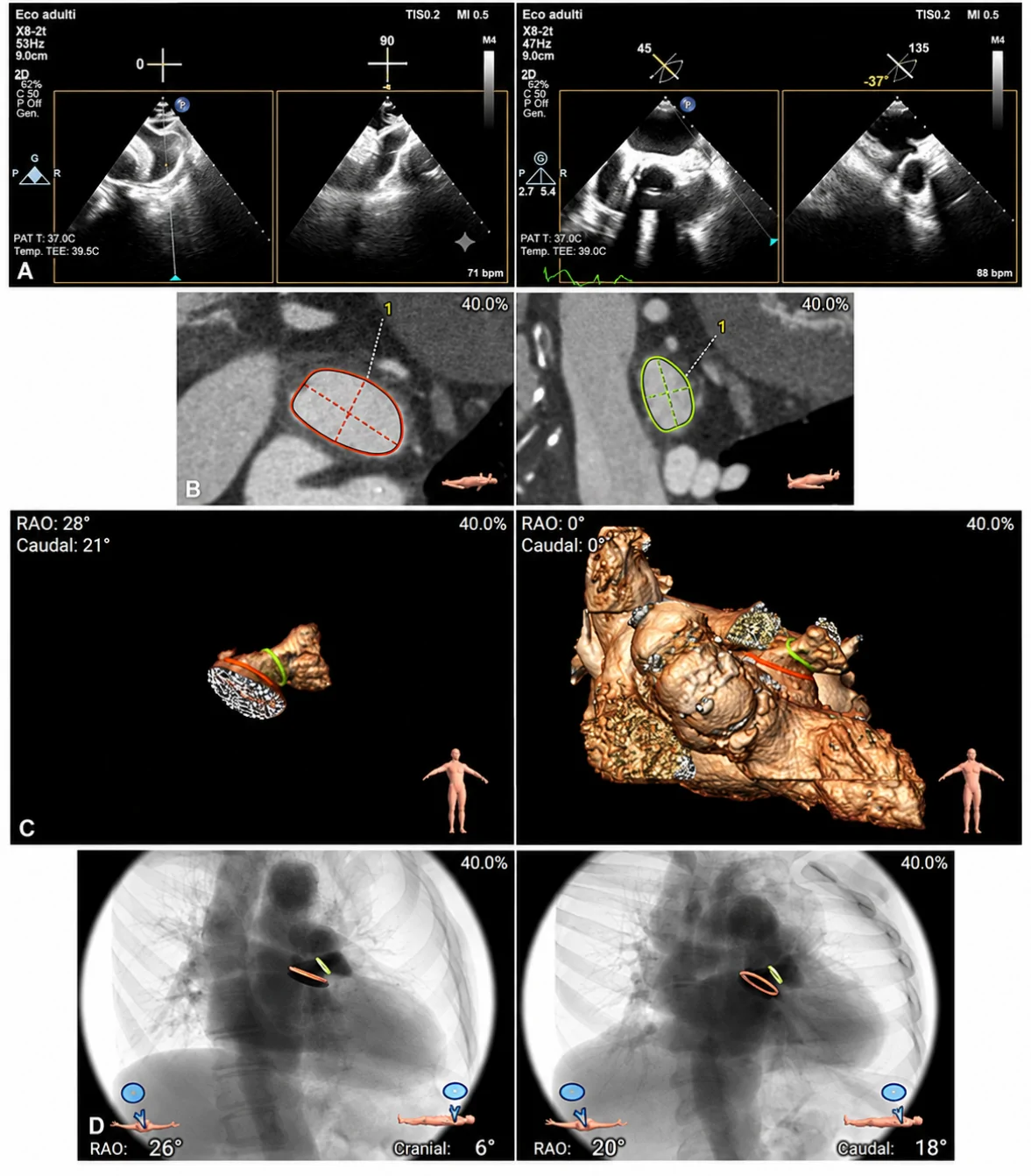
**Pre-procedural multimodality imaging for LAAC.** (**A**) Pre-procedural TEE showing suboptimal visualization of the LAA fundus, which was adequately defined only at 0°, suggesting a windsock morphology (maximum 3D diameters: 18 × 11 mm). Because of poor acoustic windows, LAA thrombus could not be definitively excluded despite multiplane and biplane imaging. (**B**,**C**) CCT confirms the absence of thrombotic filling defects and provides anatomical measurements for LAAC planning. The red contour/ring indicates the LAA ostium, the green contour/ring the more distal portion of the appendage, and the dashed lines the orthogonal diameter measurements; numeric labels are software-generated measurement markers. (**D**) CCT-derived simulated fluoroscopy showing the optimal angiographic projections for LAAC (left: RAO 26°/cranial 6°; right: RAO 20°/caudal 18°). The superimposed colored guide rings correspond to the same anatomical landmarks and facilitate procedural positioning and sizing. 3D, three-dimensional; CCT, cardiac computed tomography; LAA, left atrial appendage; LAAC, left atrial appendage closure; RAO, right anterior oblique; TEE, transesophageal echocardiography.

Beyond anatomical assessment, CCT contributes substantially to procedural planning. Three-dimensional reconstructions can identify optimal fluoroscopic working projections, facilitating device deployment and potentially reducing fluoroscopy time, radiation exposure, and contrast use [12]. More recently, CCT-based computational modeling, artificial intelligence applications and patient-specific 3D printing technologies have further expanded the role of CCT. The PREDICT-LAA trial demonstrates that AI-enabled computational modeling may improve procedural efficiency and potentially optimize procedural outcomes [22]. Similarly, feasibility studies using CCT-derived three-dimensional printed models have shown promising results for device sizing and transseptal puncture planning, supporting a future shift toward increasingly personalized LAAC procedures [23,24] (Figure 2).

### 2.4. Three-Dimensional Echocardiography and Fusion Imaging

Compared with conventional two-dimensional imaging, real-time 3D TEE (RT 3D-TEE) provides a more comprehensive assessment of the complex LAA anatomy. By enabling perimeter and area-based measurements, RT 3D-TEE improves the accuracy of ostial and landing-zone assessment and correlates more closely with the final implanted device size than conventional 2D imaging or angiography. These advantages may contribute to more accurate device sizing and optimization of device compression, potentially reducing the risk of peri-device leak [25].

Fusion imaging represents a further evolution of image guided LAAC procedures. By integrating fluoroscopy with echocardiographic or CCT dataset, fusion platforms provide simultaneous visualization of cardiac anatomy and interventional devices, facilitating key procedural steps such as transseptal puncture, device positioning and deployment [26,27].

Early studies have demonstrated the feasibility of fusion-guided LAAC, including reduced radiation exposure and contrast use [28,29]. However, current evidence is largely derived from observational studies and early clinical experience, and its impact on clinical outcomes has yet to be established.

### 2.5. Intraprocedural Imaging and Stepwise Procedural Guidance for LAAC

TEE combined with fluoroscopy remains the cornerstone of intraprocedural guidance during LAAC, although ICE is increasingly used as an alternative. The main objectives of intraprocedural imaging are to exclude LAA thrombus, confirm preprocedural measurements under appropriate loading conditions, guide transseptal puncture, assist device deployment, and verify device stability and sealing while excluding procedure-related complications such as pericardial effusion [12,26,30,31].

Following thrombus exclusion, the procedural workflow is largely standardized regardless of the device used:

- Peripheral venous access. The procedure is typically performed through the right femoral vein, which remains the preferred route for introducing the delivery system and ancillary equipment.
- Transseptal puncture. TEE or ICE is used to guide puncture of the interatrial septum and optimize catheter trajectory toward the LAA. In most cases, an infero-posterior puncture is preferred, facilitating device delivery and alignment while minimizing the risk of injury to adjacent structures.
- Device deployment. Following transseptal access, the delivery sheath and occlusion device are advanced into the LAA. Before final release, device position, stability, compression, and sealing are assessed using both fluoroscopy and echocardiography according to the PASS criteria (Position, Anchor, Size, Seal). Device anchoring is typically verified by a gentle tug test, demonstrating stable engagement of the device within the appendage. Adequate device compression is also essential, as insufficient compression (<10%) has been associated with an increased risk of PDL. When PASS criteria are not fulfilled, the device can be recaptured and repositioned prior to release. Intraprocedural TEE or ICE further allows assessment of residual peri-device flow and early procedure-related complications.
- TEE and ICE are both recommended modalities for intraprocedural guidance in LAAC. The choice between them is largely center-dependent and influenced by procedural workflow, efficiency, resource availability, complication risk, and cost considerations. TEE remains the reference standard owing to extensive operator experience and superior spatial and temporal resolution, whereas real-time 3D ICE provides adequate visualization of key structures and enables a minimally invasive approach without the need for general anesthesia or esophageal intubation. Although ICE may provide lower overall image quality, available data suggest similar procedural success and long-term thromboembolic outcomes. However, ICE may be less accurate for comprehensive LAA characterization and device sizing, supporting the continued role of preprocedural CCT when detailed anatomical assessment is required. While ICE is associated with higher upfront equipment costs, these may be partially offset by reduced use of anesthesia, shorter recovery times, and lower procedural resource utilization [32] (Table 1).

### 2.6. Postprocedural Imaging

Postprocedural Imaging is crucial after LAAC to confirm device stability and complete appendage sealing, and to detect device-related complications such as device-related thrombus (DRT), PDL, and device embolization. Before hospital discharge, transthoracic echocardiography should be performed to confirm appropriate device position and exclude pericardial effusion. When DRT or significant PDL is detected, repeat imaging at 45–90 days is recommended until resolution or stabilization is documented. Because DRT may be detected beyond the early post-implant surveillance period, additional imaging at 1 year may be considered in selected patients [33]. TEE remains the reference modality for postprocedural assessment, providing detailed evaluation of device position, residual flow, thrombus formation, and device–tissue interaction. CCT has emerged as a complementary imaging modality owing to its superior spatial resolution and greater sensitivity for detecting residual leaks and assessing device endothelialization and residual LAA patency [34]. Nevertheless, its routine use may be limited by radiation exposure, contrast administration, and availability (Table 2).

#### 2.6.1. Peri-Device Leak

PDL is the most frequent imaging finding after LAAC, although its reported incidence varies considerably according to the imaging modality, timing of assessment, and definition adopted. Historically, residual PDL ≤5 mm was considered an acceptable closure result in the pivotal WATCHMAN trials and was used as a practical criterion for discontinuation of oral anticoagulation [35]. However, more recent evidence has challenged the assumption that small residual leaks are clinically benign. Contemporary registry data and meta-analytic evidence suggest that even small TEE-detected PDLs may be associated with residual thromboembolic risk, with a graded increase in thromboembolic events across increasing TEE-based leak-size thresholds [36].

The interpretation of PDL is further influenced by the imaging modality used for surveillance. CCT is substantially more sensitive than TEE for detecting residual LAA patency and PDL [34,36]; however, the prognostic significance of small leaks detected exclusively by CCT remains less clearly established, whereas TEE-detected PDL appears to show a more consistent association with thromboembolic outcomes [36]. Therefore, PDL should not be interpreted according to size alone. Management should be individualized according to leak size and persistence, imaging modality, concomitant DRT, and the patient’s thromboembolic and bleeding risks. Larger or persistent leaks may warrant continuation or resumption of oral anticoagulation or, in selected high-risk patients unable to tolerate long-term anticoagulation, consideration of percutaneous leak closure [33,37].

#### 2.6.2. Device-Related Thrombus

DRT is an uncommon complication after LAAC, with a reported incidence of less than 4%; however, its clinical relevance is substantial. Importantly, a substantial proportion of DRT cases may be detected after the routine 45-day surveillance examination, indicating that early imaging alone may not identify all cases [38]. In the pivotal WATCHMAN trials and associated registries, protocol-mandated TEE was performed at 45 days and 12 months in all patients, with additional imaging at 6 months in the randomized trial cohorts and unscheduled imaging when clinically indicated [38]. Notably, 28% of DRTs were identified during unscheduled imaging evaluations [38]. These findings support the need for continued clinical vigilance and individualized imaging surveillance beyond the early post-implant period. Although relatively uncommon, DRT carries important prognostic implications, and is associated with an approximately four- to five-fold increased risk of ischemic stroke or systemic embolism [38,39]. Prediction of DRT remains a priority in the LAAC field given expanding procedural indications. Although several independent predictors of DRT have been identified, including hypercoagulable states, renal insufficiency, deep device implantation, pericardial effusion, and non-paroxysmal atrial fibrillation, currently available risk models show only modest predictive performance and have not been externally validated [40].

Both TEE and CCT are effective for DRT detection. However, owing to its superior spatial resolution, CCT is more sensitive for identifying subtle device abnormalities, particularly hypoattenuated thickening (HAT), which may represent either physiological endothelialization or early thrombus formation [41]. Recent CCT studies suggest that low-grade HAT is frequently a benign healing phenomenon, whereas high-grade HAT is more strongly associated with DRT and may warrant closer surveillance [42].

The management of DRT is primarily based on escalation or resumption of OAC, which is associated with high rates of thrombus resolution on follow-up imaging [43].

Although the clinical significance of HAT remains incompletely defined, increasing evidence supports an individualized approach, recognizing that not all CCT-detected abnormalities require the same therapeutic strategy [44,45] (Table 2).

## 3. Historical Randomized Evidence

Advances in multimodality imaging have substantially improved procedural planning, implantation accuracy, and postprocedural surveillance, making LAAC an increasingly reproducible and technically successful intervention [2,12,33]. Importantly, however, the randomized evidence supporting LAAC spans different technological and imaging eras, and imaging strategies have not been uniformly standardized across trials [4,5,46,47,48,49,50,51]. Procedural and imaging refinement should therefore be considered when interpreting the evolution of the evidence, while recognizing that procedural success does not necessarily translate into improved clinical outcomes. The role of LAAC ultimately depends on the evidence generated by randomized clinical trials comparing this strategy with contemporary medical therapy. The modern evidence base for LAAC began with PROTECT-AF, the first landmark randomized trial comparing Watchman implantation with warfarin in anticoagulation-eligible patients with nonvalvular AF [46,47]. LAAC proved non-inferior for the composite endpoint of stroke, systemic embolism, and cardiovascular or unexplained death, while long-term follow-up suggested reductions in hemorrhagic stroke, cardiovascular mortality, and all-cause mortality [47,48].

Nevertheless, PROTECT-AF should be interpreted within the context of the pre-DOAC era. The comparator was warfarin, procedural complications reflected the early learning curve, and the apparent long-term benefit was driven largely by reductions in bleeding and mortality rather than by superior protection against ischemic stroke. Accordingly, PROTECT-AF should be regarded primarily as a proof-of-concept trial demonstrating that mechanical exclusion of the LAA can provide stroke prevention broadly comparable to anticoagulation in selected patients [46,47,48].

PREVAIL was designed as a confirmatory trial and addressed two unresolved questions from PROTECT-AF: whether device-based appendage closure could reproduce efficacy under more rigorous scrutiny, and whether procedural safety had improved with greater operator experience [49]. As in the earlier trial, warfarin was the comparator, but PREVAIL incorporated two co-primary endpoints, including a global 18-month efficacy endpoint and a second endpoint focused on stroke or systemic embolism from day 8 onward, thereby isolating postprocedural preventive efficacy from the immediate periprocedural hazard [49]. The study met its early safety objective and achieved noninferiority for the post-7-day endpoint, but it failed to meet noninferiority for the broader 18-month composite endpoint [49]. PREVAIL remains pivotal but methodologically less definitive. On one hand, it demonstrated improved procedural safety and supported the reproducibility of LAAC in experienced centers [49]. On the other, it did not provide an unequivocal confirmation of broad clinical equivalence to warfarin, in part because of modest sample size, low event rates, and noninferiority margins that have been debated [49,50].

Taken together, PROTECT-AF and PREVAIL established feasibility, mechanistic plausibility, and long-term clinical potential, but they did not resolve the debate over the net benefit of LAAC in the era of modern anticoagulation [47,48,49].

### 3.1. DOAC-Era and Contemporary Evidence

The transition from warfarin-era trials to DOAC-era evidence is essential for defining LAAC’s current place in clinical practice. PRAGUE-17 was the first randomized study to provide a meaningful contemporary comparison by testing LAAC against DOAC therapy in patients at high risk of both thromboembolism and bleeding [50,51]. The trial showed that LAAC was noninferior for a broad composite endpoint that included cardiovascular death, stroke, systemic embolism, clinically relevant bleeding, and procedure- or device-related complications [50,51]. This trial highlights that LAAC can achieve a similar overall net clinical benefit while potentially reducing long-term bleeding burden in a particularly vulnerable population [50,51].

Its conclusions must be interpreted with appropriate restraint. The sample size was modest, the endpoint was broad and partly driven by nonfatal events, and the trial population was highly selected, which limits extrapolation to lower-risk patients or to settings with less procedural expertise. Even so, PRAGUE-17 marked an inflection point by showing that the rationale for LAAC extends beyond historical warfarin comparisons and remains relevant when judged against contemporary pharmacological therapy [50,51].

More recent studies have added both support and uncertainty to the evidence base.

The OPTION trial further expanded the contemporary evidence base by evaluating LAAC in patients undergoing catheter ablation for atrial fibrillation who remained candidates for long-term oral anticoagulation [52]. In this randomized study, 1600 patients at elevated thromboembolic risk were assigned to LAAC or oral anticoagulation and followed for 36 months. LAAC was associated with a significantly lower incidence of the primary safety endpoint of non-procedure-related major or clinically relevant nonmajor bleeding (8.5% vs. 18.1%), while meeting criteria for noninferiority for the primary efficacy endpoint of all-cause death, stroke, or systemic embolism (5.3% vs. 5.8%) [52].

OPTION is particularly relevant because it evaluated LAAC in a distinct clinical setting in which successful rhythm-control intervention does not, by itself, eliminate the indication for anticoagulation based on thromboembolic risk. However, the findings should be interpreted within the specific context of patients undergoing AF ablation and should not be automatically extrapolated to the broader AF population. Moreover, as in other contemporary LAAC trials, the study was not designed to compare alternative imaging strategies; therefore, the independent contribution of TEE- versus CCT-based planning to procedural or clinical outcomes cannot be determined [52].

CLOSURE-AF, conducted in a population at high risk of both stroke and bleeding, failed to demonstrate noninferiority of LAAC compared with best available medical therapy for the composite endpoint of stroke, systemic embolism, major bleeding, and cardiovascular death [5]. This result is important because it highlights how sensitive the value of LAAC is to patient selection, comparator therapy, and endpoint construction [5].

Importantly, the comparator in CLOSURE-AF consisted of individualized contemporary medical therapy rather than a single pharmacological strategy, reflecting real-world treatment pathways based on anticoagulation eligibility and bleeding risk [5]. Thus, LAAC was compared with flexible therapeutic pathways tailored to patient characteristics rather than with a single drug regimen.

In this setting, the neutral result is particularly informative: when LAAC is compared with individualized contemporary medical therapy rather than historical warfarin or a single DOAC strategy, its apparent benefit may narrow substantially. The study therefore shifts the discussion from a simple device-versus-drug comparison toward the more clinically relevant question of which patients derive greater net benefit from replacing tailored pharmacological care with an invasive structural strategy.

The recently published CHAMPION-AF trial further extends the discussion by evaluating LAAC in patients eligible for long-term DOAC therapy [4]. At 3 years, LAAC met criteria for noninferiority relative to DOAC therapy for the composite of cardiovascular death, stroke, and systemic embolism, while also reducing nonprocedural and overall clinically relevant bleeding. However, the observed signal toward more ischemic stroke events in the LAAC group tempers enthusiasm and prevents a simplistic interpretation of the trial as definitive evidence supporting routine first-line use [4].

The divergent results of CLOSURE-AF and CHAMPION-AF likely reflect differences in patient phenotype, comparator strategy, and endpoint structure. CLOSURE-AF enrolled an older, more clinically fragile population at high risk of both stroke and bleeding. By contrast, CHAMPION-AF studied a broader population eligible for chronic DOAC therapy, effectively testing LAAC in patients with a more favorable therapeutic profile and against a more standardized pharmacological comparator.

Differences in endpoint definition further contributed to the divergent findings. In CLOSURE-AF, the broad composite endpoint and the high comorbidity burden may have diluted any device-related advantage, whereas in CHAMPION-AF the reduction in nonprocedural bleeding contributed meaningfully to the apparent net benefit [4,5].

These two trials should therefore be read as describing distinct clinical phenotypes rather than yielding a simple yes-or-no verdict on LAAC.

An additional consideration when interpreting the randomized evidence is the parallel evolution of imaging strategies across successive eras of LAAC. Early randomized studies, including PROTECT-AF and PREVAIL, were conducted within predominantly TEE-based procedural and surveillance pathways [46,47,48,49], whereas contemporary LAAC practice increasingly incorporates CCT and multimodality imaging for preprocedural anatomical characterization, device sizing, and post-implant assessment [2,12,33]. This evolution has contributed to more accurate anatomical assessment and procedural planning; however, its impact on the differences in clinical outcomes observed across randomized trials remains difficult to isolate.

Importantly, imaging protocols were not uniformly standardized or reported across studies, and the major randomized trials were not specifically designed to compare TEE- versus CCT-based planning or to determine whether a specific imaging strategy improves long-term clinical outcomes. Moreover, differences among trials in patient selection, device generation, operator experience, comparator therapy, postprocedural antithrombotic treatment, and endpoint definitions represent important potential confounders [4,5,46,47,48,49,50,51]. Therefore, although contemporary multimodality imaging may contribute to improved procedural optimization and detection of device-related complications [2,12,33], the discrepancies in clinical outcomes across randomized trials cannot currently be attributed to differences in imaging strategy alone.

**Figure 3:**
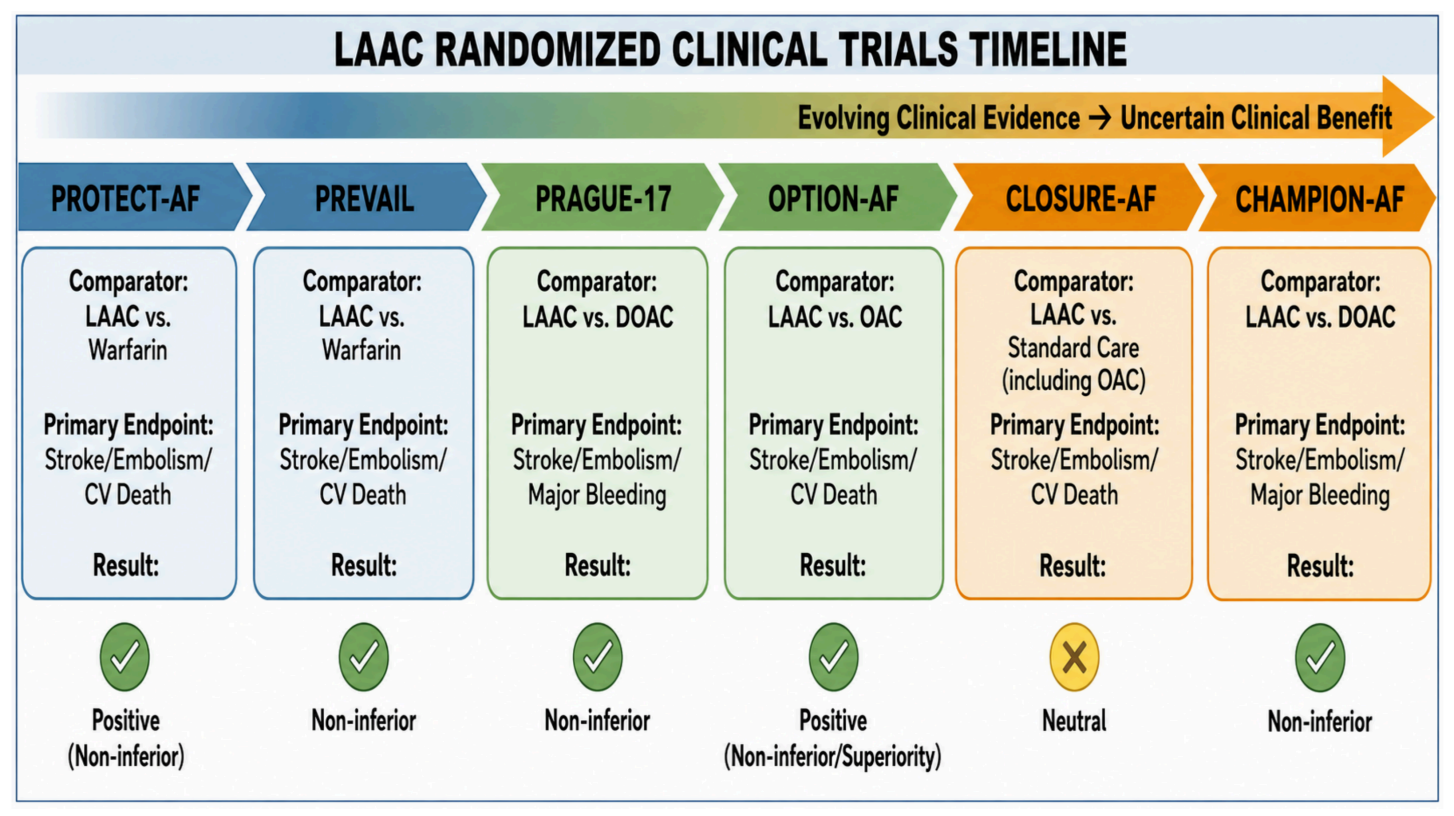
**Timeline of the major randomized clinical trials evaluating left atrial appendage closure.** The figure summarizes the evolution of randomized evidence for LAAC, including the principal comparator, primary endpoint, and overall trial result. CV, cardiovascular; LAAC, left atrial appendage closure; DOAC, direct oral anticoagulant; OAC, oral anticoagulation.

Taken together, PRAGUE-17, OPTION, CLOSURE-AF, and CHAMPION-AF suggest that the major challenge is no longer whether LAAC works, but rather to identify which patients derive a net clinical benefit from an increasingly precise procedural strategy. Accordingly, the future of LAAC is unlikely to lie in universal replacement of anticoagulation, but in increasingly refined phenotypic selection of patients most likely to benefit from a mechanical approach [4,50] (Table 3) (Figure 3).

### 3.2. Current Indications and Guideline Positioning

Current international guidelines recommend LAAC for selected patients with AF who are unsuitable for long-term oral anticoagulation because of previous major bleeding, unacceptably high bleeding risk, intolerance, or inability to maintain anticoagulant therapy [2,53,54,55].

The 2024 ESC Guidelines emphasize individualized management of AF within an integrated patient-centred approach, whereas the SCAI/HRS recommendations place particular emphasis on shared decision-making, multimodality imaging, careful patient selection, and structured post-implant follow-up [2,53,54]. Together, these documents position LAAC as a selective therapeutic option rather than a universal alternative to OAC.

However, contemporary clinical practice is evolving more rapidly than the available evidence. LAAC is increasingly being adopted in clinical scenarios that remain only partially supported by randomized data, including patients undergoing AF ablation [52], frail older adults, and individuals unwilling to continue long-term anticoagulation. While this expansion reflects increasing operator experience and procedural confidence, it also highlights the growing gap between real-world practice and the populations evaluated in randomized clinical trials.

### 3.3. Controversies and Structural Limitations

Despite an expanding body of randomized and observational evidence, several methodological limitations continue to complicate interpretation of the LAAC literature and limit generalizability. Selection bias remains a major concern, particularly in registries and randomized trials enrolling highly selected patients at the extremes of thromboembolic or bleeding risk, thereby limiting applicability to routine clinical practice.

A second major limitation is the historical weakness of head-to-head comparison with modern anticoagulation. Much of the foundational support for LAAC derives from warfarin-era trials, which remain important but no longer reflect the standard risk-benefit profile of current AF management [46,47,48,49]. Even when newer studies compare LAAC with DOACs, they often rely on broad composite endpoints that include hard clinical outcomes together with bleeding and procedure-related events, thereby making interpretation of “net benefit” more complex than a simple efficacy comparison [4,5,50,51].

**Figure 4:**
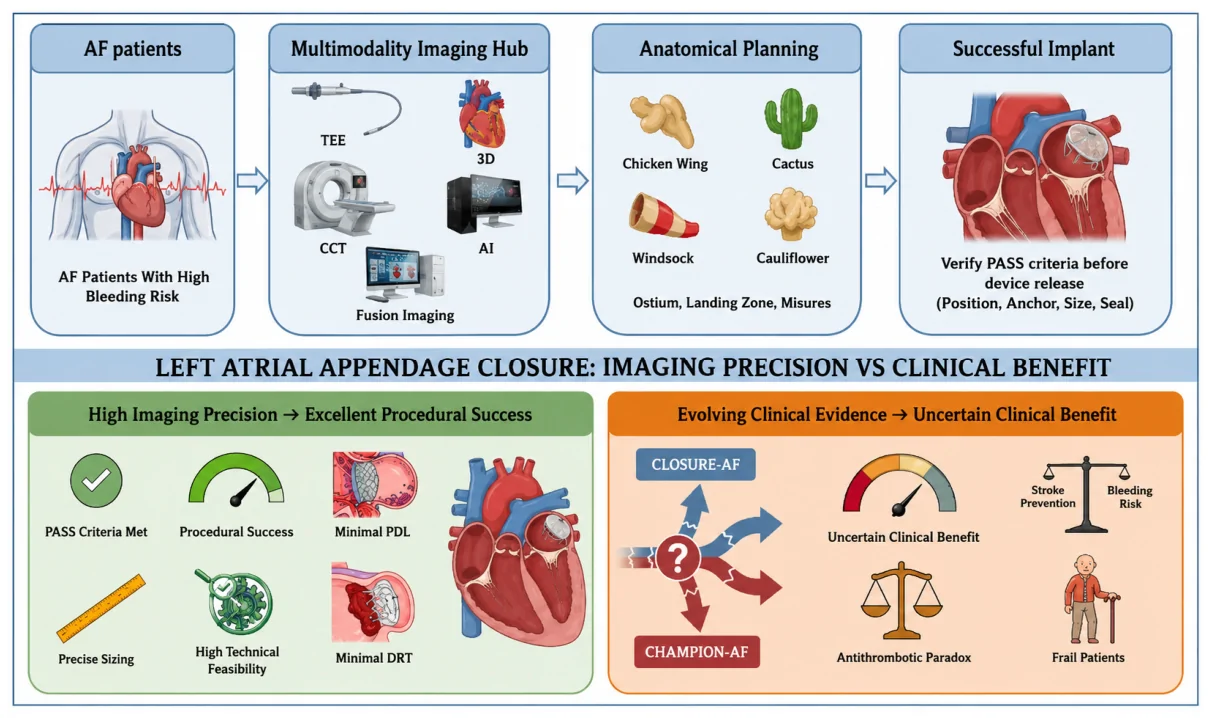
**Imaging precision versus clinical benefit in left atrial appendage closure.** The figure illustrates the continuum from patient selection and multimodality imaging to anatomical planning and successful device implantation, highlighting the contrast between increasing procedural precision and the still-evolving evidence regarding clinical benefit. 3D, three-dimensional; AF, atrial fibrillation; AI, artificial intelligence; CCT, cardiac computed tomography; DRT, device-related thrombus; PASS, position, anchor, size, and seal; PDL, peri-device leak; TEE, transesophageal echocardiography.

Finally, LAAC outcomes are intrinsically dependent on procedural expertise. Device success, peri-device leak, device-related thrombus, and procedural complications are influenced not only by patient characteristics but also by imaging quality, procedural planning, implantation technique, and structured follow-up [2,53]. Accordingly, LAAC should be viewed not as a single procedural event but as a longitudinal structural intervention whose effectiveness depends on the quality of the entire care pathway (Figure 4).

### 3.4. Complications and the Postprocedural Antithrombotic Paradox

The clinical trade-off of LAAC begins with the procedure itself, which inevitably introduces an upfront procedural risk. Pericardial effusion or tamponade, vascular complications, device embolization, and periprocedural stroke remain the principal early hazards [52,54]. Consequently, the overall clinical value of LAAC depends not only on long-term reduction in thromboembolic events but also on whether this initial procedural risk is justified for the individual patient.

Device-related complications introduce a second layer of complexity. Device-related thrombosis, residual peri-device leak, incomplete endothelialization, and the need for repeat imaging or reintervention all challenge the idea of LAAC as a simple mechanical solution [52,54]. Rather than eliminating the challenges of stroke prevention, LAAC often redistributes that complexity from chronic pharmacology to anatomical assessment, procedural execution, and structured post-implant surveillance [49,52,54]. This shift is acceptable only when the overall clinical context supports it.

The clearest paradox is that LAAC is frequently chosen to reduce long-term antithrombotic exposure, yet it almost always requires transitional antithrombotic therapy after implantation [2,47,49,50,52,54,55]. Depending on patient characteristics and device strategy, this may involve short-term anticoagulation, dual antiplatelet therapy (DAPT), or individualized regimens aimed at preventing device thrombosis and promoting endothelialization. This creates the central tension of the procedure: the patients most likely to benefit from avoiding chronic anticoagulation are often those least well suited to tolerate even short-term antithrombotic therapy [2,4].

Ultimately, the value of LAAC lies not in immediate freedom from antithrombotic therapy, but in the possibility of reducing long-term treatment burden in carefully selected patients, accepting an initial procedural risk and a period of structured post-implant management.

## 4. Conclusions

Over the past two decades, LAAC has evolved from an experimental structural intervention into an established therapeutic option for stroke prevention in selected patients with nonvalvular atrial fibrillation. Advances in multimodality imaging have played a pivotal role in this evolution, enabling increasingly accurate patient selection, procedural planning, device implantation, and postprocedural surveillance.

At the same time, contemporary randomized trials have substantially refined our understanding of the clinical role of LAAC. Rather than providing a universal answer to the question of whether LAAC should replace oral anticoagulation, studies such as PRAGUE-17, OPTION, CLOSURE-AF, and CHAMPION-AF suggest that its benefit is highly dependent on patient phenotype, comparator therapy, and individual bleeding and thromboembolic risk. Accordingly, the current challenge is no longer to demonstrate that LAAC is technically feasible, but to identify the patients most likely to derive a meaningful net clinical benefit.

An important paradox nevertheless remains. Although LAAC is intended to reduce long-term exposure to antithrombotic therapy, it still requires a period of postprocedural antithrombotic treatment and structured imaging surveillance, emphasizing that mechanical stroke prevention does not eliminate clinical complexity but rather redistributes it.

Future progress in the field is therefore unlikely to depend solely on further technological refinement. Instead, it will require more robust long-term randomized evidence, better phenotypic patient selection, and closer integration of imaging findings with clinically meaningful outcomes. Bridging the gap between imaging precision and clinical benefit remains the next major challenge in LAAC.

## Figures and Tables

**Table 1:** Current imaging techniques for LAAC: strengths, limitations, and clinical role. 2D, two-dimensional; 3D, three-dimensional; 3DP, three-dimensional printing; AI, artificial intelligence; CCT, cardiac computed tomography; LAA, left atrial appendage; LAAC, left atrial appendage closure; TEE, transesophageal echocardiography.

Imaging Modality	Main Advantages	Main Limitations	Clinical Role in LAAC
2D TEE	Real-time imaging; excellent thrombus exclusion; intraprocedural guidance; no radiation or contrast	Semi-invasive; operator-dependent; may underestimate LAA dimensions	Clinical gold standard for patient selection and procedural guidance
3D TEE	Improved LAA sizing and device selection	Limited availability and expertise	Adjunct imaging in complex anatomy
CCT	Superior 3D anatomical assessment; accurate device sizing; procedural planning; high accuracy for thrombus exclusion	Radiation, iodinated contrast, no real-time guidance	Anatomical gold standard for preprocedural planning
Fluoroscopy	Real-time visualization of catheters and device deployment	No soft tissue or thrombus assessment; radiation and contrast	Essential adjunct for device implantation
Fusion imaging	Integrates anatomy with fluoroscopy; may reduce radiation and contrast	Limited evidence and availability	Emerging tool for image-guided procedures
3DP	Patient-specific simulation and device sizing	Time, cost, limited accessibility	Investigational tool for personalized planning
AI and computational modeling	Virtual device simulation; optimized sizing and procedural planning	Limited validation and clinical implementation	Emerging technology for precision LAAC

**Table 2:** Imaging findings and their clinical implications in LAAC. CCT, cardiac computed tomography; DRT, device-related thrombus; ICE, intracardiac echocardiography; LAA, left atrial appendage; LAAC, left atrial appendage closure; PDL, peri-device leak; TEE, transesophageal echocardiography; TTE, transthoracic echocardiography.

Imaging Finding	Imaging Modality	Clinical Implication
Dense spontaneous echo contrast/low LAA emptying velocity	TEE	Marker of blood stasis and increased thromboembolic risk.
LAA thrombus	TEE, CCT	Absolute contraindication to LAAC; procedure should be deferred until thrombus resolution, usually requires anticoagulation
PDL	TEE, CCT	Residual PDL, including small TEE-detected leaks, may be associated with increased thromboembolic risk. Clinical significance and management should be individualized according to leak size and persistence, imaging modality, concomitant DRT, and the patient’s thromboembolic and bleeding risks.
DRT	TEE, CCT	Associated with an increased risk of stroke/systemic embolism; generally requires anticoagulation and follow-up imaging.
Pericardial effusion	TEE, ICE, TTE	Suggests cardiac perforation or tamponade and requires prompt management.
Device embolization	TEE, Fluoroscopy	Rare but life-threatening complication due to device instability; prompt device retrieval and reassessment of the implantation strategy are required

**Table 3:** **Major randomized trials evaluating left atrial appendage closure and corresponding imaging strategies.** Imaging protocols evolved substantially across the different trial eras and were not uniformly reported or standardized. PROTECT-AF and PREVAIL predominantly relied on TEE for procedural guidance and protocol-mandated serial surveillance, whereas contemporary trials were conducted within increasingly heterogeneous multimodality imaging pathways. Importantly, none of these trials randomized patients according to TEE- versus CCT-based procedural planning; therefore, differences in clinical outcomes across trials cannot be directly attributed to the imaging modality employed. AF, atrial fibrillation; CCT, cardiac computed tomography; DOAC, direct oral anticoagulant; ICE, intracardiac echocardiography; LAAC, left atrial appendage closure; OAC, oral anticoagulation; TEE, transesophageal echocardiography.

Study	Design and Population	Comparator	Primary Endpoint	Imaging Strategy	Key Finding	Key Methodological Limitations
PROTECT-AF [46]	Randomized trial in nonvalvular AF; patients eligible for warfarin	Warfarin	Stroke, systemic embolism, cardiovascular/unexplained death	Predominantly TEE-based. WATCHMAN implantation was performed with fluoroscopic and echocardiographic guidance; protocol-mandated TEE surveillance was performed at 45 days, 6 months, and 12 months to assess device position, sealing, and residual peri-device flow.	LAAC noninferior to warfarin; extended follow-up suggested reductions in hemorrhagic stroke, mortality, and bleeding	Warfarin comparator; first-generation device; early procedural learning curve; procedural complications; heterogeneous composite endpoint
PREVAIL [49]	Confirmatory randomized trial in nonvalvular AF	Warfarin	Co-primary endpoints: overall 18-month composite; stroke/systemic embolism from day 8 to 18 months	TEE-based procedural and surveillance strategy. Implantation was guided by fluoroscopy and TEE; ICE was rarely permitted. TEE was mandated at 45 days, 6 months, and 12 months.	Noninferiority met for the post-7-day endpoint but not for the broader composite endpoint	Few events; modest sample size; debated noninferiority margins; noncontemporary comparator
PRAGUE-17 [50]	Randomized trial in patients at high ischemic and bleeding risk	DOACs	Composite of cardiovascular, neurologic, bleeding, and procedure/device-related events	Contemporary but not defined by a single TEE- or CCT-based planning strategy. Early post-implant imaging was required, whereas longer-term serial imaging was less intensive than in PROTECT-AF/PREVAIL.	LAAC noninferior to DOACs; possible reduction in late bleeding burden	Relatively small sample; broad composite endpoint; heterogeneous devices/imaging pathways; limited generalizability
OPTION [52]	Randomized trial in patients undergoing catheter ablation for AF with elevated thromboembolic risk.	Oral anticoagulation	Primary efficacy: all-cause death, stroke, or systemic embolism; primary safety: non-procedure-related major or clinically relevant nonmajor bleeding	Contemporary multimodality imaging pathway; imaging strategy was not randomized	LAAC reduced non-procedure-related major or clinically relevant nonmajor bleeding and was noninferior to OAC for death, stroke, or systemic embolism	Specific post-ablation population; concomitant or sequential LAAC; imaging strategy not randomized; generalizability outside the AF ablation setting remains uncertain
CLOSURE-AF [5]	Randomized trial in patients at high risk of stroke and bleeding	Physician-directed best medical care, including DOACs when eligible	Stroke, systemic embolism, major bleeding, or cardiovascular/unexplained death	Contemporary multimodality practice; no uniform TEE- versus CCT-based strategy reported as a randomized component of the study. Imaging was not tested as a determinant of clinical outcome.	Noninferiority of LAAC was not demonstrated	Highly comorbid population; heterogeneous medical therapy; imaging strategy not randomized; results strongly dependent on patient selection
CHAMPION-AF [4]	Randomized trial in patients eligible for long-term DOAC therapy	DOACs	Cardiovascular death, stroke, or systemic embolism	Contemporary WATCHMAN FLX and multimodality imaging era. The trial did not randomize patients according to TEE- versus CCT-based planning; therefore, the independent contribution of imaging modality to clinical outcomes cannot be determined.	LAAC noninferior for cardiovascular death/stroke/systemic embolism; reduced non-procedure-related bleeding, with a numerical signal toward more ischemic stroke	Contemporary but selected anticoagulation-eligible population; imaging strategy not randomized; longer follow-up required to define ischemic–bleeding trade-off

## Data Availability

No new data were created or analyzed in this study. Data sharing is not applicable to this article.

## Abbreviations

The following abbreviations are used in this manuscript:

LAAC	Left atrial appendage closure
AF	atrial fibrillation
OAC	Oral anticoagulation
VKAs	vitamin K antagonists
DOACs	direct oral anticoagulants
LAA	left atrial appendage
LA	left atrium
FDA	Food and Drug Administration
TTE	transthoracic echocardiography
TEE	Transesophageal echocardiography
CCT	cardiac computed tomography
3DP	three-dimensional printing
ICE	intracardiac echocardiography
RT 3D-TEE	Real-time three-dimensional transesophageal echocardiography
DRT	device-related thrombus
PDL	peri-device leak
HAT	Hypoattenuated thickening
AI	artificial intelligence
CV	Cardiovascular
